# The Gut–Skin Axis in Atopic Dermatitis and Inflammatory Bowel Disease: Mechanisms, Microbiota, and Therapeutic Implications

**DOI:** 10.3390/jpm16090482

**Published:** 2026-09-18

**Authors:** Ezzuddin Abuhussein, Edith V. Bowers, Yukihiro Yamaguchi, Keita Nishiyama, Olivia G. Cassidy, Koichi Tsuboi, Lei Huang

**Affiliations:** 1 Pediatrics Children’s Research Institute, University of North Carolina at Chapel Hill, Chapel Hill, NC 27599, USA; 2 Department of Dermatology, University of North Carolina at Chapel Hill, Chapel Hill, NC 27599, USA; edith_bowers@med.unc.edu; 3 Department of Pediatrics, University of North Carolina at Chapel Hill, Chapel Hill, NC 27599, USA; 4 Laboratory of Animal Food Function, School of Agricultural Science, Tohoku University, Sendai 980-8572, Japan; 5 Warren Alpert Medical School of Brown University, Providence, RI 02912, USA; 6 Department of Pediatric Surgery, School of Medicine, Juntendo University, Tokyo 113-8421, Japan; 7 Faculty of Medical Sciences, Newcastle University, Newcastle upon Tyne NE2 4HH, UK

**Keywords:** gut–skin axis, atopic dermatitis (AD), inflammatory bowel disease (IBD) microbiota, dysbiosis, immune pathway

## Abstract

The gut–skin axis is a bidirectional communication network linking the gastrointestinal tract, skin, microbiota, and immune system. As major barrier organs, the gut and skin harbor complex microbial communities that contribute to tissue homeostasis, immune regulation, and protection from environmental insults. Increasing evidence indicates that dysbiosis of the gut and skin microbiota is associated with inflammatory diseases through interconnected microbial, metabolic, and immune pathways. Clinical observations further reveal strong associations between gastrointestinal disorders, including inflammatory bowel disease (IBD) and celiac disease, and cutaneous manifestations. Atopic dermatitis (AD) is characterized by epithelial barrier dysfunction, immune dysregulation, microbial imbalance, and environmental influences. Patients with AD frequently exhibit reduced gut microbial diversity, depletion of beneficial short-chain fatty acid (SCFA)-producing bacteria, and enrichment of potentially pathogenic microorganisms. Cutaneous dysbiosis, particularly expansion of *Staphylococcus aureus*, can further impair the skin barrier and sustain inflammation. Microbial metabolites, including SCFAs, tryptophan-derived aryl hydrocarbon receptor ligands, and bile acid metabolites, may mediate gut–skin communication by regulating epithelial integrity, immune tolerance, and inflammatory signaling. IBD is also increasingly recognized as a systemic disorder involving alterations in skin microbiota and cutaneous immunity. Intestinal inflammation may disrupt immune tolerance to skin commensals and promote cutaneous inflammation through cytokine signaling and immune-cell trafficking, while emerging evidence suggests reciprocal skin-to-gut effects. Epidemiologic, genetic, and clinical studies indicate an association between AD and IBD, potentially reflecting shared genetic susceptibility, barrier dysfunction, dysbiosis, and immune pathways. This review summarizes current evidence linking the gut–skin axis to AD and IBD.

## 1. Introduction

Inflammatory bowel disease (IBD), including Crohn’s disease (CD) and ulcerative colitis (UC), is a chronic, recurrent, inflammatory condition that arises from a combination of genetic, environmental, intestinal microflora, epithelial barrier dysfunction, and immune mechanisms [1,2]. Although CD and UC share many pathogenic mechanisms, they exhibit distinct pathological patterns. CD can affect any region of the gastrointestinal (GI) tract and is characterized by discontinuous “skip” lesions and transmural inflammation, which predisposes patients to strictures, fistulas, and abscesses. In contrast, UC is restricted to the colon and rectum and typically produces continuous inflammation beginning in the rectum and extending proximally, with inflammation predominantly involving the mucosa and submucosa. Histologically, CD may demonstrate deep fissuring ulcers and non-caseating granulomas, whereas UC is characterized by mucosal ulceration, crypt abscesses, and pseudopolyps. These differences contribute to distinct clinical manifestations and complications and reflect partially divergent immune mechanisms, including prominent Th1/Th17-associated inflammation in CD and atypical Th2/Th9-associated responses in UC [1,2]. IBD can also cause systemic inflammation and extraintestinal manifestations affecting organs and systems outside the digestive tract in 25% to 50% of patients.

IBD pathogenesis reflects genetic susceptibility (e.g., NODs, ATG16L1, IL23R), epithelial barrier dysfunction, and intestinal dysbiosis, which drive the dysregulated Th1/Th17 (CD) and atypical Th2 (UC) responses described above [3]. Management has evolved from corticosteroids, 5-aminosalicylates, and immunomodulators toward targeted biologics (anti-TNF agents, vedolizumab, IL-12/23 or IL-23-selective inhibitors) and oral small molecules (JAK inhibitors, S1P modulators), with surgery remaining important for refractory or complicated disease [4].

The gut–organ axis should be regarded as a bidirectional communication system between the GI tract and other organs [5,6]. The gut–organ axis is formed by a complex network of interactions between the GI tract and its microbiota with distant organs that is best understood as an interconnected network of individual two-link connections. Intestinal bacteria, their metabolites, and immune cells traffic to distant organs and modulate their function. Short-chain fatty acids (SCFAs), bile acids, tryptophan metabolites, lipopolysaccharides (LPS), cytokines, and immune cells are common molecules involved in the communication between the gut and other organs. Moreover, neural and endocrine connections contribute to the bidirectional nature of the gut–organ axis. Thus, intestinal dysbiosis and barrier dysfunction can lead to a state of increased inflammation that involves the brain [7,8], liver [9,10], lungs [11,12,13], kidneys [14,15], and pancreas [16]. On the other hand, malfunction of these organs can affect the intestines and change the microbiome and immunity of the gut. These findings provide an integrated view of mechanisms by which IBD can cause extraintestinal manifestations. They also suggest that treatments that target the microbiota, gut immunity, and the production of beneficial metabolites by the microbiota might have beneficial effects beyond the intestine.

The concept of the gut–skin axis has emerged as a central framework for understanding the bidirectional interactions between the GI tract, skin, microbiota, and immune system. The gut and skin are major barrier organs that are constantly exposed to environmental insults and depend on resident microbiota to maintain their integrity. Microbial imbalance in either the gut or skin can promote local and systemic inflammation. Environmental stimuli, especially air pollutants, cause oxidative stress and inflammation by disrupting the gut microflora. Redox mechanisms may contribute to gut dysbiosis, inflammation, and downstream effects on the skin, underscoring the role of oxidative stress and microbial communication in gut–skin axis dysfunction [17].

Associations between GI and cutaneous diseases are well-supported by a previous study [18]. Notably, 10% to 25% of patients with GI diseases such as IBD and celiac disease also experience skin pathology, including psoriasis (characterized by scaly plaques and hypertrophic skin lesions) and cutaneous ulcers [18,19]. Celiac disease, which is characterized by intestinal malabsorption, commonly co-presents with dermatitis and psoriasis as cutaneous manifestations, whereas patients with rosacea (a chronic inflammatory skin condition that causes reddened skin and a rash) frequently exhibit small intestinal bacterial overgrowth (SIBO) [20]. Peutz-Jeghers Syndrome, which is characterized by GI polyposis and malignancy, often features perioral hyperpigmentation as a common cutaneous manifestation [19]. Additionally, IBD patients frequently experience skin manifestations such as skin ulcers, vasculitis, hair loss, erythema folliculitis, and psoriasis [18,19], with some of these conditions linked to the severity of the patient’s GI inflammation [21]. 

One important mechanism of gut-to-skin communication involves microbial metabolites, which have been revealed to mediate the dialogue between the two organs. Indeed, beneficial metabolites like SCFAs and tryptophan derivatives mediate immune tolerance, induce regulatory T-cell differentiation, and induce anti-inflammatory cytokines IL-10 and TGF-β, among other processes. Some microbial metabolites may also influence keratinocytes and epidermal barrier regulation after entering the bloodstream. In other words, harmful metabolites such as phenolics could promote skin inflammation and disruption of the epidermal barrier. In summary, the manipulation of microbial metabolites as a treatment approach for skin diseases like atopic dermatitis (AD) via probiotics and prebiotics seems promising [22].

Recent advances in knowledge have gone beyond the classical paradigm of gut-to-skin interaction by developing a bidirectional model of the gut–skin axis. Numerous review articles exist regarding the gut–skin axis and skin diseases, such as atopic dermatitis [23,24,25,26,27]. In contrast, there are few papers discussing the impact of the gut–skin axis on enteritis [28,29]. While the impact of gut microbiota on skin integrity is well established, recent findings indicate that signaling pathways related to innate immunity, vitamin D receptors, and aryl hydrocarbon receptor (AhR) could play a role in maintaining skin and gut homeostasis. Interestingly, it was found that ultraviolet B (UVB) irradiation of the skin is associated with favorable changes in gut microbiome and physiology [30]. These findings suggest that the gut and skin should be viewed as interconnected organs within a shared immune–microbial network rather than as isolated systems [31].

From a personalized medicine perspective, differences in microbiome composition, host genetics, and immune responses may contribute to variation in disease susceptibility, severity, and therapeutic response. Integrating these features may therefore enable improved patient stratification, biomarker development, and selection of microbiota- or immune-targeted therapies. Unlike prior reviews focused primarily on gut microbiota or gut-to-skin effects in individual diseases, the present review considers evidence across both AD and IBD and highlights the emerging role of eosinophils in bidirectional gut–skin communication.

## 2. Methods

We have clarified the literature search and selection strategy used for this review. The review was developed through a comprehensive literature retrieval focusing on the gut–skin axis, with particular attention to the relationship between gut microbiota, immune and skin inflammation, and translational dermatology. We searched PubMed/MEDLINE, Google Scholar, and Web of Science for relevant peer-reviewed literature published from 1985 through 2026.

The search strategy incorporated combinations of keywords and, where applicable, MeSH terms related to the major themes of this review, including “gut–skin axis,” “microbiome,” “microbiota,” “inflammatory bowel disease,” and “atopic dermatitis,”. Representative search combinations included (“gut–skin axis”) AND (“microbiome” OR “microbiota”), (“inflammatory bowel disease”) AND (“microbiome” OR “microbiota”), (“inflammatory bowel disease”) AND (“atopic dermatitis”), and (“atopic dermatitis”) AND (“microbiome” OR “microbiota”).

The literature considered in this review included peer-reviewed original research articles, reviews, observational studies, clinical studies, clinical trials, animal studies, and relevant in vitro investigations. Particular emphasis was placed on studies providing empirical evidence for bidirectional communication among the gut and skin; alterations in the intestinal or cutaneous microbiota; immune and inflammatory pathways; microbial metabolites and metabolic signaling; and microbiota-targeted or other translational interventions relevant to inflammatory skin diseases. Relevant case reports were also considered when they provided clinically informative observations pertinent to the topics discussed.

The retrieved literature was screened for relevance to the scope of the review, and eligible studies were organized and critically evaluated according to their contribution to the mechanistic and translational framework. Literature that was unrelated to the gut–skin axis or the dermatological conditions and mechanisms discussed in the review was not considered. Rather than representing a formal systematic review or meta-analysis, this article was designed as a comprehensive narrative review integrating findings from diverse experimental and clinical literature. We have revised the Section 2 accordingly to make the search strategy, scope, and nature of the evidence synthesis more transparent.

## 3. Microbiota

### 3.1. Gut Microbiota

Gut microbiota refers to a diverse and dynamic community of microorganisms, including bacteria, viruses, fungi, and archaea, that inhabit the GI tract and function as an integrated ecosystem within the host. Far from being a mere passive collection of microbes, it acts as a functional organ that co-evolves with the host, playing an essential role in digestion, immune regulation, metabolism, and the maintenance of overall physiological homeostasis [32,33].

Gut microbiota contributes to nutrient processing and the regulation of metabolism by breaking down dietary components that are otherwise indigestible, such as complex carbohydrates and dietary fiber, and producing bioactive metabolites that influence host physiology. These metabolites include SCFAs, bile acid derivatives, and amino acid-derived metabolites, which modulate energy balance, epithelial function, and immune signaling pathways [33,34]. Furthermore, the gut microbiota is involved in the synthesis of vitamins, such as vitamin K and B vitamins, thereby serving as a metabolic partner in host physiological processes [32].

One of the primary functions of gut microbiota is its role in the development and regulation of the immune system. Microbial communities constantly interact with innate and adaptive immune cells, promoting immune system maturation and enabling responses to pathogens while maintaining immune tolerance toward harmless antigens. A disruption of this balance can lead to immune-mediated diseases, including chronic inflammation and IBD [33,35].

Importantly, microbial metabolites act directly on immune cell populations, such as dendritic cells, macrophages, and T cells, thereby linking microbiota composition to systemic immune responses [33]. The gut microbiota also plays a crucial role in maintaining the integrity of the intestinal barrier and ecological stability. Beneficial microbes compete with pathogens, reinforce epithelial defense mechanisms, and support mucosal barrier function, thereby preventing microbial translocation and inflammation [32]. At an ecosystem level, the structure of the microbiota is shaped by ecological processes such as host-mediated environmental filtering, nutrient availability, colonization history, and inter-microbial competition. The interplay of these processes determines the assembly and stability of the microbiota in adulthood [34]. Furthermore, microbial communities are not uniformly distributed within the gut; instead, they occupy spatially distinct niches, influenced by oxygen gradients, the mucus layer, and site-specific physiological characteristics, to form a highly structured biogeographical system [36].

The gut microbiota exerts systemic effects that extend beyond the intestinal tract, influencing distant organs and disease pathology. Through host–microbiota signaling pathways, such as the gut–brain and gut–immune axes, bacterial metabolites and signaling molecules can impact metabolism, cardiovascular health, neurobehavioral functions, and inflammatory responses [32,33]. Regarding disease associations, an imbalance in the gut microbiota is strongly linked to IBD such as CD and UC; reduced microbial diversity and altered compositional profiles are recognized as consistent features in the pathogenesis of these conditions [37]. In the specific case of CD, bacterial translocation into mesenteric adipose tissue may contribute to the formation of “creeping fat,” suggesting a link between microbiota alterations and processes such as tissue remodeling and fibrosis [38].

Importantly, the gut microbiota exhibits a high degree of plasticity and responsiveness to environmental factors. This plasticity is particularly pronounced during early infancy, when the microbiota becomes established, yet it remains subject to change across all age groups. Diet is a primary determinant of microbiota composition, while factors such as antibiotics, stress, sleep patterns, and environmental exposures can also profoundly influence its community structure and function [32,34]. This plasticity underpins the growing interest in the gut microbiota as a novel therapeutic target. Research is currently underway on interventions, including probiotics, prebiotics, dietary therapies, and fecal microbiota transplantation, targeting a wide range of conditions, from IBD and necrotizing enterocolitis to immune-mediated diseases [33,39,40].

### 3.2. Skin Microbiota

The human skin microbiota is a complex and dynamic ecosystem composed of bacteria, fungi, viruses, and microeukaryotes that work in concert to maintain skin homeostasis and contribute to host defense. In healthy individuals, this microbial community is shaped by distinct skin niches, which are influenced by local physiological conditions such as sebum levels, moisture content, pH, and temperature, resulting in the formation of site-specific microbiota. On healthy skin, resident microorganisms are not merely a passive layer of microbes; rather, they function as integral components of skin biology, contributing to the maintenance of barrier function, the education of the immune system, and resistance against pathogen colonization [41,42].

Advances in sequencing technologies, particularly amplicon and shotgun metagenomic analyses, have enabled detailed characterization of the skin microbiome at both community and strain levels, revealing significant inter-individual variation and ecological complexity [41]. These studies highlight that microbial composition is shaped not only by anatomical site but also by host-specific factors such as genetics, age, sex, immune status, and environmental exposures [42]. Crucially, the skin microbiome is highly dynamic, reflecting constant interactions among microbial species and between microbes and host tissues; these communities form tightly regulated ecosystems that contribute to skin resilience and homeostasis [43]. While skin health relies on balanced microbial interactions, dysbiosis is associated with various skin diseases. In AD, reduced microbial diversity and the overgrowth of *Staphylococcus aureus* are characteristic findings, with disease severity closely linked to microbial imbalances and strain-specific virulence factors [44,45]. Furthermore, disruptions of microbiota in early life can influence immune system development and potentially increase susceptibility to AD and food allergies, underscoring the importance of the skin microbiome during the period of immune maturation [46]. Similar patterns of dysbiosis have been implicated in other skin conditions, such as acne, psoriasis, seborrheic dermatitis, and alopecia areata, underscoring the broad relevance of microbiome alterations in cutaneous pathology [47].

Recent research has shifted the focus beyond mere compositional changes to emphasize the functional and ecological interactions within the skin microbiome. Commensal bacteria, such as *Staphylococcus epidermidis*, *Cutibacterium acnes*, and the genus *Roseomonas*, inhibit pathogens through antimicrobial activity and the suppression of virulence, while simultaneously modulating the host immune response via interactions with keratinocytes and immune cells [45,48]. These interactions involve the production of bacteriocins, proteases, and metabolites that suppress pathogens like *Staphylococcus aureus* and regulate the expression of virulence genes, revealing an intricate network of microbial competition and cooperation [48]. In this context, the skin microbiome functions as an ecological barrier that actively defends against pathogen invasion while maintaining immune homeostasis.

## 4. AD and Gut–Skin Axis

AD is now known as a complex immune-mediated systemic disease with intricate relationships between genetic predisposition, impaired skin barrier function, immune dysfunction, dysbiosis, and metabolism. Disruption of the gut–skin axis plays an important role in the development of chronic inflammation, sensitization, and immune dysfunction typical of AD [49,50,51,52]. Peng et al. revealed enrichment of some gut microbiota (e.g., *Bacteroides plebeius*, *Bacteroides thetaiotaomicron*, *Bacteroides xylanisolvens*, and *Parabacteroides merdae*) in mild-to-moderate pediatric AD [53].

An emerging trend in the recent literature is considering gut microbial dysbiosis as a pivotal factor in the development of AD. Many patients with AD have abnormal gut microbiomes, which are defined by an increased presence of pathogenic microflora, namely *Clostridium difficile*, *Escherichia coli*, *Staphylococcus aureus*, as well as lower levels of commensal microorganisms like *Bifidobacterium*, *Bacteroides*, and other producers of SCFAs [54]. The impairment of intestinal barrier function, systemic immune response, and chronic cutaneous inflammation may be caused by intestinal dysbiosis. At the same time, the skin microbiota of patients with AD is characterized by decreased diversity with overgrowth of *S. aureus*. Thus, intestinal and skin dysbiosis appear to be interrelated processes.

Microbial metabolites are considered essential mediators involved in the interaction between gut microbes and the skin immune system. SCFAs, including butyrate, acetate, and propionate, help maintain skin epithelial barrier integrity, regulate Treg development, and inhibit the activity of signaling pathways involved in triggering an inflammatory reaction. Thus, low levels of SCFAs in AD can be regarded as one of the contributing factors to immune tolerance disruption and inflammatory reactions [54,55]. Besides SCFAs, tryptophan AhR agonists and bile acid-dependent FXR/TGR5 signaling pathways are increasingly recognized as key modulators of epithelial stability and immune regulation [24,55]. Altered production of microbial metabolites may trigger abnormal Th2-driven inflammation accompanied by IL-4/IL-13 pathway stimulation, contributing to the impairment of skin barrier function observed in AD [55]. Metagenomics-, metabolomics-, and transcriptomics-based multi-omics studies have recently identified unique endotypes of AD, defined by microbial and metabolic characteristics. Specifically, *Bacteroides*-dominant enterotypes were associated with lipopolysaccharide-induced inflammation, while *Prevotella*-containing ones were associated with AhR activation and skin regeneration [24]. Therefore, AD can be considered a heterogeneous disease with multiple endotypes.

Recent studies have also highlighted the importance of immune-cell trafficking along the gut–skin axis. Aberrant migration of immune cells between intestinal and cutaneous tissues may facilitate propagation of inflammation across distant organs [56]. Under inflammatory conditions, gut-primed immune cells may acquire altered homing receptor expression and migrate to the skin, thereby contributing to chronic dermatologic inflammation [57,58,59,60,61,62,63,64]. Conversely, cutaneous immune activation may influence intestinal immune responses through reciprocal trafficking mechanisms [56,65]. Dysregulated migration of dendritic cells, T cells, and other immune populations may therefore represent an important mechanism linking intestinal and skin inflammation beyond microbial and metabolic communication alone. However, the molecular signals governing immune-cell reprogramming, tissue-specific homing, and dendritic-cell egress remain incompletely understood.

The growing recognition of microbiota-mediated immunometabolic regulation has stimulated interest in microbiome-targeted therapeutic strategies in AD. Probiotics, particularly multi-strain formulations dominated by *Lactobacillus* and *Bifidobacterium* species, have demonstrated modest but relatively consistent clinical benefits in pediatric AD, including improvements in disease severity, pruritus, and inflammatory biomarkers [26]. Mechanistically, probiotics may enhance SCFA production, suppress toll-like receptor (TLR)2/TLR4-NF-κB inflammatory signaling, and promote IL-10- and TGFβ-mediated immune tolerance. Nevertheless, clinical evidence remains heterogeneous due to differences in probiotic strains, dosages, study populations, and outcome measures. Consequently, probiotics are currently considered adjunctive therapies rather than replacements for standard dermatologic treatments.

Experimental studies further support the therapeutic relevance of microbial metabolites in AD. Colon-targeted delivery of butyrate using butyrylated *Smilax glabra* starch (BSGS) significantly alleviated cutaneous inflammation in murine AD models by restoring intestinal barrier integrity, modulating gut microbiota composition, and suppressing NF-κB signaling [66]. BSGS treatment increased beneficial microbial taxa such as *Bacteroides* and *Prevotellaceae* while reducing inflammation-associated bacteria, thereby reinforcing the concept that modulation of gut microbial metabolism can improve systemic and cutaneous immune homeostasis. These findings highlight the therapeutic potential of microbiota-derived metabolites and colon-targeted metabolic interventions for chronic inflammatory skin diseases.

Emerging precision-medicine approaches aim to integrate host genetics, microbiome composition, metabolomics, and immune profiling to improve disease stratification and therapeutic selection in AD [24]. Future therapeutic strategies may include genotype-guided biologics, engineered probiotics, bacteriophage therapy, postbiotics, fecal microbiota transplantation, and precision nutrition approaches targeting individualized microbial and metabolic abnormalities. However, substantial challenges remain, including heterogeneity among studies, limited longitudinal microbiome data, insufficient mechanistic validation, and the need for standardized multi-omics profiling. Future investigations integrating gut and skin microbiome analyses, immune-cell trafficking studies, metabolomic profiling, and long-term clinical outcomes will be essential for establishing microbiome-based precision therapies.

## 5. IBD and Gut–Skin Axis

IBD is increasingly recognized as a systemic disease that affects not only the gut microbiota but also the skin microbial ecosystem. Direct analysis of skin bacterial communities has revealed that patients with CD and UC possess skin microbiota profiles distinct from those of healthy individuals, providing evidence that dysbiosis extends beyond the GI tract [67]. The most pronounced changes were observed in the retroauricular crease, where CD patients exhibited significantly higher microbial diversity compared to UC patients and healthy controls. Common microbial shifts observed in both CD and UC included an increase in *Corynebacterium* and *Pseudomonas*, alongside a decrease in commensal genera such as *Cutibacterium*, *Actinomyces*, *Lawsonella*, *Prevotella*, and *Streptococcus*. However, disease-specific characteristics were also identified; *Corynebacterium*, *Bacteroides*, and *Methylobacterium methylo-rubrum* were abundant in CD, whereas *Cutibacterium*, *Delftia*, and *Psychrobacter* were relatively more prevalent in UC [67]. These findings suggest that distinct subtypes of IBD are associated with unique skin microbiota profiles. Evidence from experimental studies indicates that intestinal inflammation can directly alter the host immune response to skin-resident microbes. Research using mouse models of colitis demonstrated that intestinal inflammation impairs immune tolerance to the skin commensal *Staphylococcus epidermidis* by reducing commensal-specific regulatory T cells (Tregs) in both the skin and skin-draining lymph nodes, thereby increasing neutrophilic inflammation in the skin [68]. These changes were driven by IL-1-dependent signaling and increased damage-associated molecular patterns (DAMPs)^+^ T-cell trafficking between gut-associated and skin-associated lymphoid tissues. This study provides mechanistic evidence that intestinal inflammation remodels cutaneous immune homeostasis and alters host–microbiota interactions at a distant barrier site, thereby supporting a functional gut–skin axis [68].

Genetic studies further substantiate the biological link between intestinal and cutaneous inflammation. Genome-wide analyses have revealed a substantial shared genetic architecture between IBD and psoriasis, including evidence of positive genetic correlation and causality, as well as 43 shared susceptibility loci enriched in immune-related tissues such as blood, spleen, and lymphocytes [28]. Similarly, Mendelian randomization analyses demonstrated that genetically predicted IBD increases the risk of hidradenitis suppurativa (HS), whereas HS does not appear to exert a causal effect on IBD; this suggests that intestinal inflammation may promote the development of certain inflammatory skin diseases, rather than the reverse [69,70]. Meanwhile, Dokoshi et al. identified a previously unrecognized “skin-to-gut axis” in mice, wherein skin injury actively disrupts intestinal immunity and the gut microbiota [65]. They demonstrated that skin damage, caused by physical wounds or elevated dermal hyaluronidase activity, triggers the systemic release of hyaluronic acid fragments acting as DAMPs. These signals elicit a robust intestinal epithelial response characterized by the upregulation of genes associated with mucus and antimicrobial substances (specifically the Muc2 and Reg3 families), thereby reinforcing the host’s basal defense mechanisms in the colon.

The link between IBD, skin disorders, and imbalance in microbial flora is further highlighted by research into follicular skin diseases such as HS. A systematic review concluded that, in addition to alterations in gut and skin microbiota, shared inflammatory cytokine networks and T-cell responses likely contribute to the frequent comorbidity of HS and IBD [71]. Specific microbial groups, including *Enterococcus* and *Veillonella*, are associated with disease severity, and dietary interventions that alter gut microbiota composition may also influence disease outcomes. These findings are consistent with a potential role for microbiota-mediated immune dysregulation in linking intestinal and cutaneous inflammation [71]. Although changes in the skin microbiota may have potential as biomarkers of disease status or treatment response, further studies are needed to determine their clinical utility, including their ability to predict treatment-related complications in IBD. Patients with CD who subsequently developed cutaneous adverse events associated with anti-TNF agents exhibited characteristic patterns in their skin microbiota profiles prior to the initiation of treatment. Specifically, these patterns were characterized by a reduction in commensal bacteria such as *Cutibacterium* and *Staphylococcus hominis*, alongside an increase in genera including *Corynebacterium*, *Micrococcus*, *Enhydrobacter*, *Dietzia*, *Gemella*, and *Kocuria* [67]. Although longitudinal analyses did not confirm consistent microbiota changes during the process of lesion formation, these pre-treatment microbiota characteristics suggest that skin dysbiosis may predispose certain patients to cutaneous toxicity during biologic therapy.

## 6. IBD and AD: The Bidirectional Relationship

A large body of epidemiologic, genetic, and clinical evidence suggests a complex and partially bidirectional association between AD and IBD, although the magnitude and even the direction of this relationship vary across study design, population, and analytical approach (Table 1). Early population-based cohort data from Denmark indicated that adult AD was not associated with an increased risk of incident IBD after adjustment for confounders (HR for CD 0.69, UC 0.94) [72], suggesting that previously observed associations might reflect prevalence or detection bias rather than true causality. Similarly, a nationwide Taiwanese cohort found no increased long-term risk of IBD in patients with AD [73], reinforcing the possibility that adult-onset AD may not independently drive IBD development in some populations.

Several large observational cohorts and meta-analyses have reported a consistent positive association between AD and IBD. A landmark German cohort study demonstrated increased risk of CD and UC in patients with AD, with risk ratios of 1.34 and 1.25, respectively [74]. More recent large-scale databases, including the UK-based THIN cohort, showed a 34% increased risk of IBD in adults with AD and a 44% increased risk in children, with a clear severity-dependent relationship [80]. Similarly, a Scandinavian birth cohort confirmed that early-life AD (by age 3) is associated with later IBD (aHR 1.46), affecting both CD and UC [81]. A US-based nationwide database further supported this association, reporting approximately twofold increased odds of IBD in individuals with AD [82]. A Taiwanese case–control study even reported a markedly elevated odds ratio (OR 5.73), particularly in moderate-to-severe AD [85], although the wide confidence intervals and design suggest possible inflation of effect size.

Meta-analyses have generally confirmed a modest but statistically robust association between AD and IBD. A 2025 meta-analysis including over 61 million participants found a pooled odds ratio of 1.37 for IBD, with consistent associations for CD (OR 1.51) and UC (OR 1.33), albeit with substantial heterogeneity in subtype analyses [83,84]. Earlier meta-analyses similarly demonstrated bidirectional associations, with roughly 30–80% increased relative risks depending on direction and outcome definition [75,76]. Collectively, these data support a reproducible epidemiologic link, though effect sizes remain modest and heterogeneous.

Genetic and causal inference studies, however, provide a more nuanced interpretation of directionality. One Mendelian randomization analysis suggested a causal effect of AD on IBD (OR 1.11) [78], whereas another found the opposite direction, implicating IBD as a potential causal factor for AD rather than vice versa [77]. These conflicting results highlight the limitations of genetic instruments and heterogeneity in genome-wide association study (GWAS) datasets, leaving the causal direction unresolved. Additional mechanistic hypotheses propose shared immune dysregulation across Th2 (AD) and Th17/Th1 (IBD) pathways, supporting the idea that both diseases may arise from overlapping immune architecture rather than strict causality in one direction.

Importantly, AD may also influence the clinical course of IBD rather than merely its incidence. A multicenter observational study found that patients with both AD and IBD had shorter biologics-free survival, particularly in ulcerative colitis, suggesting more aggressive disease requiring earlier escalation of therapy (HR~1.83) [79]. This supports the concept that AD may serve as a marker of systemic inflammatory burden or immune dysregulation influencing disease severity.

Therapeutically, the intersection of AD and IBD is further highlighted by paradoxical and overlapping treatment responses. Anti-TNFα therapy for IBD has been associated with the development of de novo AD, likely reflecting immune deviation toward Th2 responses [86]. In such cases, targeting IL-4/IL-13 signaling with dupilumab has been shown to be effective without exacerbating IBD activity. Additionally, emerging dual-biologic strategies targeting both Th2 and Th17 pathways have demonstrated feasibility and clinical benefit in small cohorts of patients with overlapping AD, psoriasis, and IBD [87], underscoring the shared immunological network underlying these conditions.

Finally, interpretive analyses emphasize that although relative risks are often statistically significant, the absolute risk increase is small, with very high numbers needed to harm, suggesting limited clinical impact at the individual level [88]. This is particularly important given the low baseline incidence of IBD in the general population. Therefore, while AD is consistently associated with IBD at a population level, routine clinical screening for IBD in patients with AD is not currently supported by evidence.

Despite the overall evidence supporting an association between AD and IBD, several limitations should be considered when interpreting these findings. Most epidemiologic studies are observational and therefore remain susceptible to selection bias, surveillance bias, and residual confounding. Patients with AD may have more frequent healthcare encounters and specialist evaluations, potentially increasing the likelihood of IBD detection compared with individuals without AD. Conversely, patients with established IBD may have greater healthcare utilization and increased opportunities for recognition of concomitant dermatologic disease. Medication exposure may represent another important source of confounding, as corticosteroids, immunomodulators, biologic therapies, and other systemic treatments can affect the clinical manifestations and detection of both AD and IBD. Shared referral patterns between dermatology and gastroenterology, differences in healthcare access, and variation in diagnostic practices across countries and healthcare systems may further influence reported associations. Although several studies attempted to adjust for healthcare utilization and other potential confounders, unmeasured or residual confounding cannot be excluded. Furthermore, study quality varies across this literature. Several of the meta-analyses cited above assessed methodological quality using the Newcastle–Ottawa Scale [75,83,84], while the primary cohort studies differ in diagnostic method (administrative or claims-based diagnosis versus clinician-confirmed diagnosis) and degree of covariate adjustment. Differences in study design, quality, and population characteristics suggest that the magnitude of the association should be interpreted cautiously. Mendelian randomization studies provide additional evidence regarding potential causality, but their findings have been directionally discordant, emphasizing that the causal relationship between AD and IBD remains incompletely established.

## 7. Roles of Eosinophils in IBD and AD Along the Gut–Skin Axis

Eosinophils are critical effector cells in type 2 inflammation and contribute to both cutaneous and GI immune responses. In AD, elevated circulating cell numbers of eosinophils and eosinophil-derived granule proteins have long been associated with disease activity, while eosinophils accumulate in lesional skin and release cytotoxic mediators that can contribute to tissue injury and persistent inflammation. Type 2 cytokines, particularly IL-5, promote eosinophilopoiesis, recruitment, activation, and prolonged survival, providing a mechanistic link between Th2 responses and eosinophil-mediated tissue damage in AD [89,90]. Therapeutic strategies targeting type 2 cytokine pathways may also modify eosinophil recruitment and activity in AD, further supporting the clinical relevance of eosinophil-associated inflammatory networks [90].

Eosinophils also have prominent roles in the GI tract beyond their classical function as inflammatory effector cells. GI tract eosinophils play a role in mucosal homeostasis, epithelial barrier regulation, tissue development, repair, and protection against pathogens, as well as interactions with other immune and stromal cells. Their functional heterogeneity likely means that intestinal eosinophils can have protective or pathological effects depending on tissue infiltration and activation state [91,92]. In allergic GI tract inflammation, eosinophils and mast cells have been established as important mediators of mucosal responses, and eosinophil-derived mediators might serve as potential biomarkers of intestinal allergic inflammation [93]. These findings are applicable to IBD, where eosinophils can be activated in the intestinal mucosa; however, eosinophils are not established as key drivers of CD or UC. They support the general idea that eosinophils participate in intestinal immune regulation and may contribute to inflammatory processes in susceptible individuals [91,93].

The role of eosinophils in the gut–skin axis is demonstrated by experiments showing that an inflammatory stimulus at one epithelial site may also affect the recruitment and/or activation of eosinophils at a remote site [94]. For example, inhibition of LTB4 in OVA-sensitized mice profoundly reduced eosinophil infiltration into the skin and intestine, whereas anti-IL-5 strongly suppressed infiltration of eosinophils into the skin, indicating that the recruitment of eosinophils to the intestine and to the skin can be controlled by common inflammatory pathways [94].

The link between intestinal eosinophils, microbiota, and AD is of particular interest. The gut microbiome regulates immune development and systemic immune homeostasis, whereas intestinal dysbiosis can influence skin immunity and barrier function [27,95]. Experimental AD models provide evidence that cutaneous allergen exposure can induce eosinophil accumulation and inflammatory changes in the small intestine. In one model, epicutaneous allergen challenge increased intestinal eosinophils, whereas eosinophil deficiency or depletion of intestinal bacteria attenuated inflammatory responses in both the intestine and skin, supporting a bidirectional relationship among cutaneous inflammation, intestinal eosinophils, microbiota, and skin disease [96]. Thus, intestinal eosinophils may function not only as downstream effectors but also as intermediaries through which intestinal immune and microbial signals influence systemic and cutaneous inflammation.

A similar mechanism in a psoriasis model has been demonstrated and adds another piece of evidence for eosinophil-mediated skin-to-gut communication. TLR7 activation via imiquimod led to eosinophil degranulation in the small intestine, disruption of intestinal barrier integrity, and induction of intestinal inflammatory responses. Transfer of eosinophils corrected the impairment in intestinal inflammation in eosinophil-deficient mice, which had decreased skin and intestinal inflammation [97]. This indicates that TLR7-dependent activation can convey messages from the skin to the intestine, but the inflammation in the intestine can also boost the cutaneous disease [97]. Although it is psoriasis rather than AD or IBD that is at the center of the study, it is still valuable insight into the mechanisms of how eosinophils can participate in a bidirectional dialogue between the two organs.

## 8. Personalized Medicine

The gut–skin axis is a rapidly evolving field, but several important limitations currently restrict our understanding of the relationship between AD, IBD, microbiota, and immune regulation. Addressing these challenges will be essential for translating mechanistic discoveries into clinical applications.

### 8.1. Predominantly Associative Rather than Causal Evidence

Most clinical studies demonstrate a correlation between microbial dysbiosis and disease phenotypes, rather than a direct causal link. Alterations in gut or skin microbiota may not be primary drivers of pathology but rather consequences of inflammation, medication use, dietary changes, or disease severity. While animal models support a causal relationship between microbiota and immune dysfunction, translating these findings to human disease remains challenging. Future research must integrate prospective cohort studies with mechanistic investigations to determine whether microbial alterations precede disease onset or arise as a secondary consequence of inflammation. Large-scale birth cohorts may prove particularly valuable in identifying early-life microbial factors that increase the risk of developing AD or IBD.

### 8.2. Heterogeneity Among Patients and Studies

AD and IBD are highly heterogeneous conditions characterized by diverse clinical phenotypes, genetic backgrounds, environmental exposures, and treatment histories. Microbiome studies often report inconsistent results due to variations in patient populations, sampling methods, sequencing platforms, and bioinformatic analysis techniques. Consequently, a universal microbial signature has yet to be identified for either disease. Integrating metagenomics, meta-transcriptomics, metabolomics, proteomics, epigenomics, and single-cell sequencing promises a more comprehensive understanding of host–microbe interactions. Such approaches could reveal key microbial metabolites, signaling pathways, and immune cell populations that mediate gut–skin crosstalk. These advances may pave the way for personalized nutritional interventions, microbiota-targeted dietary strategies, and tailored probiotic therapies aimed at restoring immune homeostasis and improving clinical outcomes [98].

### 8.3. Limited Integration of Multi-Organ Microbiome Analyses

Many studies have focused on investigating either the gut microbiota or the skin microbiota in isolation. Simultaneous analysis of microbial communities across the gut, skin, and other mucosal surfaces within the same individual remains relatively rare. Consequently, the mechanisms governing microbial exchange and interactions among these barrier organs are not yet fully understood. Future research should aim to identify biomarkers capable of predicting gut and skin inflammation; such biomarkers include microbial taxa, blood cytokines, microbial metabolites, markers of epithelial barrier function, and immune cell signatures. These biomarkers could facilitate early diagnosis and patient stratification.

### 8.4. Insufficient Longitudinal and Interventional Studies

Many studies are cross-sectional, providing only a snapshot of microbiota composition at a single point in time. Longitudinal studies tracking microbiota dynamics before disease onset, during progression, and after treatment are limited. Similarly, there have been relatively few controlled clinical trials evaluating microbiota-targeted interventions in patients with co-occurring AD and IBD.

### 8.5. Limitations of Experimental Models

Animal models do not fully recapitulate the complexities of the human microbiome, environmental exposures, diet, or immune responses. Furthermore, since the physiology of mouse skin and the intestinal tract differs significantly from that of humans, the validity of extrapolating certain findings to humans may be limited. Human intestinal and skin organoids, organ-on-a-chip systems [99], and integrated gut–skin co-culture platforms could serve as more physiologically relevant models for studying host–microbe interactions and evaluating therapeutic interventions.

### 8.6. Investigation of Microbial Metabolites as Therapeutic Targets

SCFAs, AhR ligands, secondary bile acids, indole derivatives, and other microbiota-derived metabolites are promising therapeutic candidates. SCFAs support epithelial barrier integrity, promote Treg differentiation, and suppress inflammatory signaling [54], whereas AhR and bile acid-dependent FXR/TGR5 signaling regulate epithelial stability and immune response [24]. Probiotics may act further upstream by enhancing SCFA production, suppressing TLR2/TLR4-NF-κB signaling, and promoting IL-10- and TGF-β-mediated immune tolerance [26]. Microbiota-targeted strategies can therefore act either by modifying microbial communities or by delivering specific microbial metabolites [26]. Metabolite-directed approaches may offer more predictable effects because the active compounds and mechanisms are better defined, whereas ecological approaches such as probiotics may have broader but more variable effects. The two strategies may also be complementary.

### 8.7. Elucidation of Immune-Cell Trafficking Between Barrier Organs

Further research is needed to elucidate how T cells, innate lymphoid cells, dendritic cells, macrophages, and B cells traffic between gut-associated lymphoid tissue and skin-associated lymphoid tissue. Unraveling these pathways could lead to the identification of novel mechanisms linking intestinal inflammation and skin inflammation.

### 8.8. Exploring Epigenetic Regulation of the Gut–Skin Axis

An accumulating body of evidence suggests that microbial metabolites can influence DNA methylation, histone modifications, and chromatin accessibility in immune and epithelial cells. Future research is needed to elucidate how epigenetic regulators (e.g., TET enzymes, DNA methyltransferases, histone deacetylases, and non-coding RNAs) contribute to gut–skin crosstalk and disease susceptibility.

## 9. Conclusions

**Figure 1:**
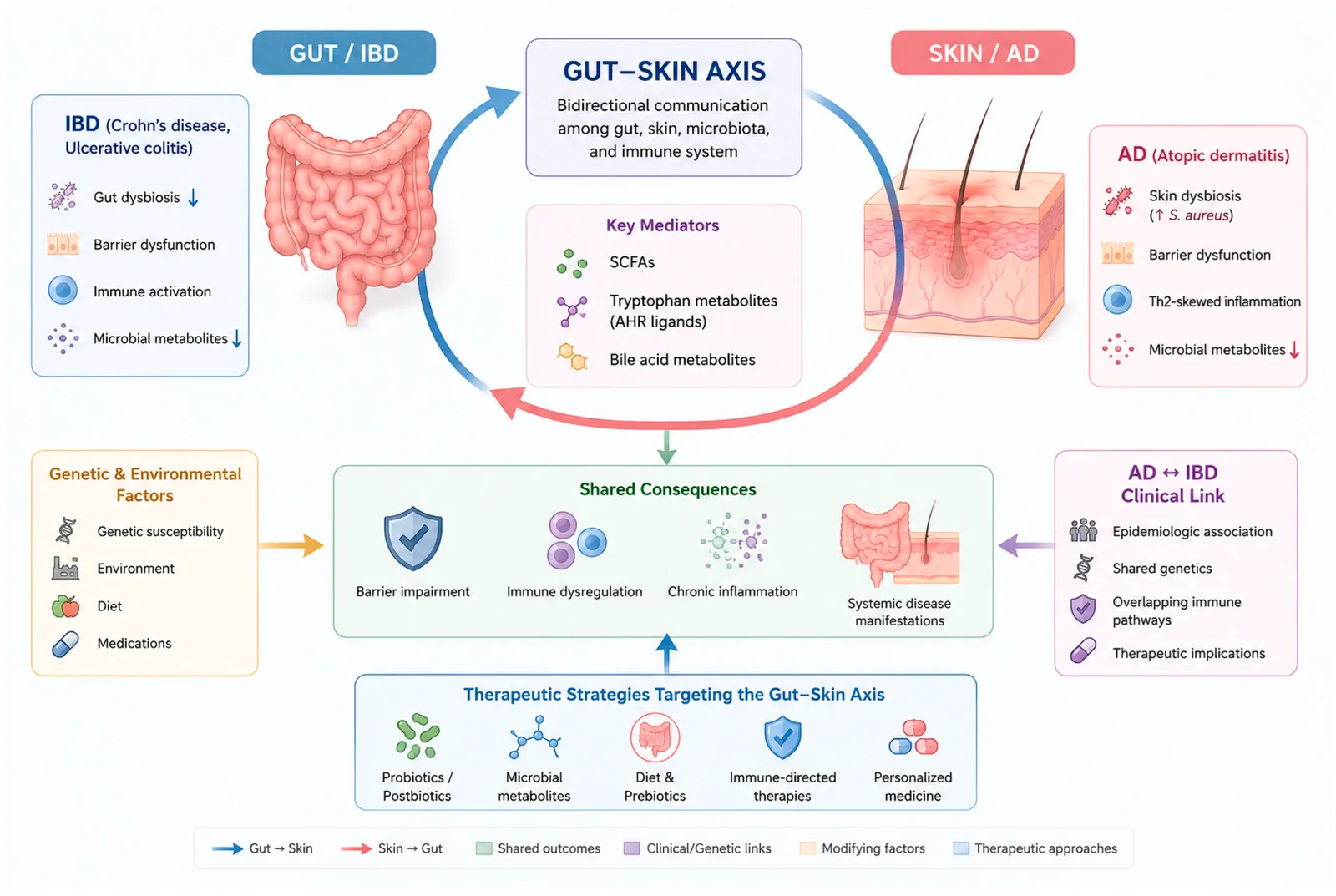
**Proposed mechanisms linking atopic dermatitis (AD) and inflammatory bowel disease (IBD) through the gut–skin axis.** The gut and skin communicate through microbial, immune, and metabolic pathways. Dysbiosis, barrier dysfunction, altered microbial metabolites, and immune dysregulation contribute to the pathogenesis of both AD and IBD. Bidirectional signaling between the gut and skin promotes chronic inflammation and may explain the epidemiologic and mechanistic association between these disorders. Targeting the gut–skin axis represents a promising therapeutic strategy for inflammatory diseases affecting both organs.

Under physiological conditions, the intestinal epithelium barrier and mucosal immune system establish tolerance to commensal microbes while providing protection to the host from pathogens. The integrity of the intestinal barrier, appropriate interactions with the microbiota, and protective immune responses are altered by genetic and environmental factors in IBD. Dysregulated intestinal immunity leads to structural damage and loss of function of the intestinal barrier, which, in turn, promotes altered microbiota composition and dysregulated immune responses to the microbiota. Therefore, IBD results from a complex interaction between the host’s immune system, intestinal barrier, and microbiota, as well as environmental factors, rather than an isolated abnormal immune response [1,100,101]. Therapeutic strategies for IBD have consequently evolved from nonspecific suppression of inflammation toward mechanism-based and targeted therapies [2,100,102] (Figure 1).

Future research should move beyond descriptive microbiome analyses toward mechanistic, longitudinal, and interventional studies that integrate microbial, metabolic, immunologic, and epigenetic data. A deeper understanding of the gut–skin axis will likely reveal novel biomarkers and therapeutic targets, ultimately enabling personalized medicine approaches for patients with AD, IBD, and other immune-mediated inflammatory disorders.

## Figures and Tables

**Table 1:** Epidemiologic and genetic evidence for the association between IBD and AD.

Study	Population/Sample Size	Design	Association Examined	Effect Estimate (95% CI)	Direction/Principal Finding
Schmitt et al., 2016 [74]	German nationwide health-insurance cohort; 655,815 individuals ≤ 40 years, including 49,847 with AD	Retrospective cohort	AD → incident IBD	CD: RR 1.34 (1.11–1.61); UC: RR 1.25 (1.03–1.53)	Positive association; AD associated with increased risk of incident CD and UC
Egeberg et al., 2017 [72]	Denmark; 7032 adults with AD vs. 3,587,974 population controls	Nationwide registry-based cohort	AD → incident IBD	CD: HR 0.69 (0.34–1.30); UC: HR 0.94 (0.61–1.43)	No significant association with incident CD or UC, despite higher baseline IBD prevalence among patients with AD
Lee et al., 2020 [75]	95,291,110 participants from 10 observational studies	Systematic review and meta-analysis	Bidirectional AD ↔ IBD	IBD → AD: OR 1.39 (1.28–1.50); AD → IBD prevalence: OR 1.35 (1.05–1.73); incident IBD: RR 1.46 (0.98–2.17)	Overall bidirectional association; evidence for incident IBD was less definitive
Shi et al., 2020 [76]	14 eligible observational studies	Systematic review and meta-analysis; Newcastle–Ottawa Scale used for quality assessment	Bidirectional AD ↔ IBD	IBD → AD: RR 1.83 (1.39–2.40); CD → AD: RR 2.06 (1.61–2.64); UC → AD: RR 1.66 (1.23–2.24); AD associated with 48% higher likelihood of IBD, 44% of CD, and 38% of UC	Bidirectional positive association; both IBD and its major subtypes were associated with AD
Weng et al., 2021 [73]	Taiwan; 36,400 patients with AD and 364,000 matched controls	Nationwide retrospective cohort	AD → incident IBD	Cumulative IBD incidence: 0.047% vs. 0.047%; *p* = 0.973	No significant association between AD and subsequent IBD
Gu et al., 2022 [77]	IBD GWAS: 34,652 subjects; AD GWAS: 212,036 subjects	Two-sample bidirectional Mendelian randomization	IBD ↔ AD	IBD → AD: β = 0.159, *p* = 0.01 (IVW); reverse MR: *p* = 0.43	Possible IBD → AD causal effect; no evidence for AD → IBD in this analysis
Meisinger & Freuer, 2022 [78]	AD GWAS: 10,788 cases/30,047 controls; IBD outcomes from UK Biobank and an independent European cohort	Two-sample bidirectional Mendelian randomization	AD ↔ IBD	AD → IBD: OR 1.11 (1.04–1.18) in combined analysis; IBD → AD: not significant	Possible AD → IBD causal effect; opposite direction from Gu et al.
Kim et al., 2023 [79]	Korea; 61 patients with IBD + AD vs. 122 matched IBD controls	Multicenter retrospective observational study	AD → IBD clinical course	All IBD: HR 1.83 (1.02–3.27); UC: HR 3.50 (1.07–11.48); CD: HR 1.54 (0.72–3.30)	AD associated with shorter biologics-free survival, particularly in UC
Chiesa Fuxench et al., 2023 [80]	UK THIN; 409,431 children and 625,083 adults with AD plus matched controls	Population-based matched cohort	AD → incident IBD	Children: IBD HR 1.44 (1.31–1.58); CD 1.74 (1.54–1.97); adults: IBD 1.34 (1.27–1.40); CD 1.36 (1.26–1.47); UC 1.32 (1.24–1.41)	Positive association; risk increased with AD severity
Lerchova et al., 2024 [81]	Sweden/Norway prospective birth cohorts; 83,311 children	Prospective population-based birth cohort	Early childhood AD → later IBD	IBD: aHR 1.46 (1.13–1.88); CD: 1.53 (1.04–2.26); UC: 1.78 (1.15–2.75)	Positive association between AD at age 3 and subsequent IBD
Joel et al., 2024 [82]	US All of Us Research Program; 296,440 adults	Cross-sectional observational study	AD ↔ prevalent IBD	IBD: aOR 1.97 (1.75–2.21); CD: 1.91 (1.64–2.22); UC: 2.02 (1.74–2.35)	Positive association with prevalent IBD, CD, and UC
Yu et al., 2024 [83]	61,190,816 participants from 8 longitudinal cohort studies	Meta-analysis of longitudinal studies	AD → incident IBD	IBD: OR 1.37 (1.31–1.43); CD: 1.51 (1.31–1.76); UC: 1.33 (1.13–1.56)	Positive pooled association; substantial heterogeneity for CD and UC
Wan & Yang, 2025 [84]	61,190,816 participants from 8 retrospective cohort studies	Meta-analysis	AD → IBD	IBD: OR 1.37 (1.31–1.43); CD: 1.51 (1.31–1.76); UC: 1.33 (1.13–1.56)	Positive pooled association; overall effect was modest, with heterogeneity for disease subtypes
Tseng et al., 2026 [85]	Taiwan; 300 incident IBD cases and 2400 matched controls	Nationwide case–control study	AD → IBD	OR 5.73 (1.69–19.48)	Positive association; markedly elevated point estimate with wide confidence interval

Footnote: Effect estimates are reported as presented in the original studies. Because study designs, populations, outcomes, and analytic methods differed, effect estimates should not be interpreted as directly comparable across studies.

## Data Availability

No new data were created or analyzed in this study. Data sharing is not applicable to this article.

## Abbreviations

The following abbreviations are used in this manuscript:

AD	Atopic dermatitis
AhR	Aryl hydrocarbon receptor
ATG16L1	Autophagy-related 16-like 1
BSGS	Butyrylated *Smilax glabra* starch
CD	Crohn’s disease
CD4	Cluster of differentiation 4
DAMPs	Damage-associated molecular patterns
DNA	Deoxyribonucleic acid
FXR	Farnesoid X receptor
GI	Gastrointestinal
GWAS	Genome-wide association study
HR	Hazard ratio
HS	Hidradenitis suppurativa
IBD	Inflammatory bowel disease
IL-1	Interleukin-1
IL-4	Interleukin-4
IL-5	Interleukin-5
IL-10	Interleukin-10
IL-12	Interleukin-12
IL-13	Interleukin-13
IL-23	Interleukin-23
IL-23R	Interleukin-23 receptor
JAK	Janus kinase
LTB4	Leukotriene B4
Muc2	Mucin 2
NF-κB	Nuclear factor kappa-light-chain-enhancer of activated B cells
NOD2	Nucleotide-binding oligomerization domain containing 2
OR	Odds ratio
OVA	Ovalbumin
Reg3	Regenerating family member 3
RNA	Ribonucleic acid
SCFA	Short-chain fatty acid
SIBO	Small intestinal bacterial overgrowth
S1P	Sphingosine-1-phosphat
TET	Ten-eleven translocation
TGF-β	Transforming growth factor-beta
TGR5	Takeda G protein-coupled receptor 5
Th1	Helper cell 1
Th2	Helper cell 2
Th9	Helper cell 9
Th17	Helper cell 17
THIN	The health improvement network
TLR2	Toll-like receptor 2
TLR4	Toll-like receptor 4
TLR7	Toll-like receptor 7
TNF	Tumor necrosis factor
TNFα	Tumor necrosis factor-alpha
Tregs	Regulatory T cells
UC	Ulcerative colitis
UVB	Ultraviolet B
VDR	Vitamin D receptor
